# The Acute Effect of a Novel Miso-Type Sauce, Enhanced with a Carotenoid-Rich Extract from Fruit By-Products, on Postprandial Biomarkers of Oxidative Stress and Inflammation

**DOI:** 10.3390/nu14061316

**Published:** 2022-03-21

**Authors:** Olga Papagianni, Eleni Delli, Melina-Eleni Vasila, Thomas Loukas, Athanasios Magkoutis, Charalampia Dimou, Haralampos C. Karantonis, Antonios E. Koutelidakis

**Affiliations:** 1 Human Nutrition Unit, Laboratory of Nutrition and Public Health, Food Science and Nutrition Department, University of the Aegean, 11472 Myrina, Greece; olga3_pap@yahoo.gr (O.P.); fnsm20010@fns.aegean.gr (E.D.); fns15064@fns.aegean.gr (M.-E.V.); chadim@aegean.gr (C.D.); 2 Outpatιent Clinic, 11472 Myrina, Greece; tloukas2002@yahoo.com (T.L.); tmagoutis@gmail.com (A.M.); 3 Laboratory of Food Chemistry, Biochemistry and Technology, Food Science and Nutrition Department, University of the Aegean, 11472 Myrina, Greece; chkarantonis@aegean.gr

**Keywords:** postprandial bioactivity, novel miso-type sauce, bioactive components, carotenoid-rich extract, fruit by-products, functional sauce, metabolic biomarkers

## Abstract

Several fruit by-products may exert a beneficial role on oxidative stress and inflammation modulation, providing essential bioactive components, such as polyphenols and carotenoids. Recently, the potential bioactivity of miso has been reported. The aim of this dietary intervention–clinical study was to evaluate the acute effect of a novel, functional miso-type sauce based on legumes, on postprandial biomarkers of oxidative stress and inflammation. In this randomized, cross-over design, intervention–clinical trial, 14 healthy volunteers, aged 20–30 years old, consumed a rice meal rich in fat and carbohydrates (258 g), containing a legume-based sauce. After a 1-week washout period, the same subjects consumed the same meal, containing the novel fermented miso-type sauce, enhanced with 50% carotenoid-rich, fruit peel extract. Differences in postprandial total plasma antioxidant capacity according to the FRAP method, serum lipids, glucose, uric acid levels, and antithrombotic activity in platelet-rich plasma were evaluated before, 30 min, 1.5 h, and 3 h after consumption. The results showed that, in comparison to the control group, consumption of the novel sauce resulted in a significantly increased total plasma antioxidant capacity 3 h after consumption (*p* < 0.05). In addition, we observed a significant attenuation of triglycerides concentration increase in the last 1.5 h in the functional group (*p* < 0.05). A significant decrease in serum aggregation was found at 30 min and 3 h after functional sauce intake in comparison with the baseline (*p* < 0.05). Finally, LDL-cholesterol concentrations were significantly reduced 3 h after the functional meal consumption, in comparison with baseline values (*p* < 0.05). The remaining biomarkers did not show statistically significant differences (*p* > 0.05). Further investigation is needed in order to validate the current results.

## 1. Introduction

Unhealthy eating habits, insufficient physical activity, and extensive exposure to chemicals are just some of the lifestyle changes that several factors, such as the COVID-19 pandemic, have caused worldwide [1]. Taking into account the need to satisfy new consumers’ requirements for immune protection and disease prevention, innovative functional foods production may be a key factor in the food industry’s repertoire [1,2].

Under these circumstances, the recovery and utilization of valuable bioactive compounds, after the exploitation of food by-products, is one of the most sustainable approaches [3]. The need arises for their utilization through food science applications, such as the extraction of bioactive ingredients, including carotenoids, polyphenols, and other phytochemicals. In this way, by-products of the food industry can be used as raw materials for the production of significant value-added compounds, such as polyphenols and carotenoids, which may have the ability to reduce oxidative stress and inflammatory biomarkers, and therefore they can be used for novel functional food production [4,5].

Scientific evidence indicates that the suppression of postprandial lipemic and glycemic responses, in combination with the oxidative stress improvement, could be achieved by the consumption of bio-functional compounds and may protect from metabolic diseases [6,7]. Therefore, the supplementation of meals high in fat and carbohydrates with plant bioactive compounds may be a key strategy for improving postprandial hyperglycemia, hyperlipidemia, and oxidative stress [8]. Previous interventional studies support the possible metabolic effect of bioactive compounds, derived from food by-products. Nevertheless, the data about the potential postprandial bioactivity of functional foods, enhanced with specific bioactive compounds, are still insufficient [8,9].

There are indications that soy fermented products consumption may exert a beneficial role on lipidemic, glycemic profile, and oxidative blood status [10]. It is notable that fermentation may have a beneficial effect on the antioxidant content of various substrates, such as barley, wheat, and rice bran [11]. Thus, the enhancement of fermented miso-type sauces with dietary antioxidants may contribute to increased bioactivity of the final fermentation product, thanks to the beneficial action of hydrolytic enzymes found in the *Aspergillus* sp. yeast, which is used for the fermentation [11,12]. However, no previous studies have investigated the possible postprandial effect of a meal containing an innovative miso-type sauce based on legumes, enhanced with carotenoids, derived from fruit by-products.

The aim of this study was to investigate how the enhancement of a Greek miso-type sauce based on legumes, with carotenoids from fruit peel extracts, may affect biomarkers of postprandial lipemia, glycemia, oxidative stress, and other biomarkers in healthy volunteers.

## 2. Materials and Methods

### 2.1. Study Participants

This cross-over, nutritional intervention–clinical study, started on 16 October 2021 and was completed on 16 December 2021. The study was carried out in accordance with the Declaration of Helsinki and was approved by the Ethics committee of the University of the Aegean (no 10/30.09.2021), in order to ensure the suitable interventional practices.

A total of 20 volunteers were initially screened, performing appointments from October 2021 to November 2021. Eventually, 14 subjects, 6 men and 8 women, aged 20–30 years, with admission data from the screening, were recruited from Lemnos, Greece, to voluntarily participate in this study. Exclusion criteria included factors that could lead to unstable conclusions or could affect participants’ overall health, as follows: age over 30 years (in order to ensure the homogeneity of the sample); antioxidant supplementation during the last 6 months; history of chronic diseases, e.g., type I and II diabetes (HbA1c > 5%); heavy smoking (>5 cigarettes/day); abnormal body mass index (BMI < 18.5 kg/m^2^ or >25 kg/m^2^); past and current alcohol abuse (>40 g/day); food allergies, especially for *Lathyrus* sp. crops; and a hematological and biochemical profile beyond normal values (cholesterol > 6.8 mM, triglycerides > 2.8 mM, glucose > 6.11 mM).

### 2.2. Data Collection

All volunteers were initially tested by personal appointment. A medical history questionnaire was used and demographic characteristics and physical activity levels were also recorded. Using a brief-type, self-administrated questionnaire, nutritional attitudes, such as the frequency of consumption of antioxidant-rich foods, as well as general habits, such as smoking and alcohol consumption, during the preceding 6-month period were evaluated. Subsequently, anthropometric measurements were performed, especially measurements of height, weight, and body composition, using a suitable body composition monitor (Tanita SC 330 P, Tokyo, Japan). Before the trial sessions began, all participants underwent biochemical blood tests in collaboration with external physicians. All participants were informed about the ultimate goal of this study, the confidentiality of the data obtained, and the voluntary nature of participation. Furthermore, after agreement with the above and knowing about the possibility of leaving at any time, all subjects signed a consent form.

### 2.3. Treatments

**Figure 1:**
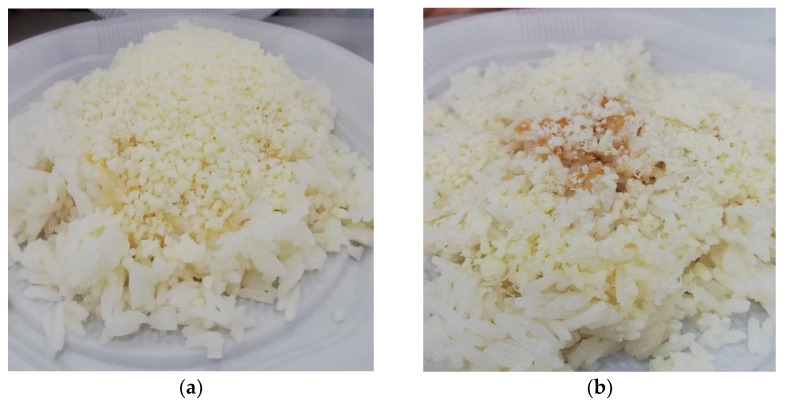
(**a**) Control meal; (**b**) functional meal, before consumption.

The two experimental meals, control and functional, weighed a total of 258 g and had the same meal basis, containing 175 g of white jasmine rice (Agrino, Greece), 45 g of Amfilochia pecorino cheese (Amfigal, Amfilochia, Greece), 20 g of unsalted butter (Lurpak, Unsalted, Arla Foods Hellas, Athens, Greece), and 18 g of a legume-based (for the control meal) or the novel miso-type sauce (for the functional meal). The novel miso-type sauce was produced as follows: 100 g of solid material (50% cereal milling by-products at a ratio of 2:1:1 of Mavragani wheat and corn by-product streams mixed with 50% chickpeas of third sorting) inoculated with 0.05% (*w/w*) *A. oryzae* spores and incubated for 48 h at 28 °C [13]. The fermented mass was mixed with water to achieve a 15% final humidity of the medium. The mixture was left at 4 °C during the initial 10 days of bioprocessing. Then, the miso product was mixed with a salt solution to achieve a final concentration of the final product equal to 15% *w/v* in NaCl and the temperature was increased to 35 °C until the 30th day of the fermentation. The salt solution contained 15 g of NaCl while the remaining 85 mL consisted of water–ethanol extracts (mixture containing 30% carrot, 30% orange, 20% apple, 10% banana, and 5% kiwi fruit by-product extracts). The extraction took place under optimized conditions, using ethanol: water (60:40, *v/v*) as a solvent, at 50 °C for 140 min, under 250 W ultrasound power with an initial solid to liquid concentration equal to 0.05 mg/mL. The control sauce was a mixture of 50% Greek legume paste (Afkos: chickpeas, 1:1, *w/w*) and 50% boiled water. Figure 1 shows the control (a) and functional (b) meals, prior to their consumption. The dietary composition of the test meals is shown in Table 1.

### 2.4. In Vitro Functional Sauce Preliminary Determinations

In order to calculate the nutritional value of the novel, functional test meal, in vitro preliminary determinations of the composition of the innovative, functional miso-type sauce in total proteins, phenolic components, and carotenoids was performed by Bradford, Folin–Ciocalteu, and total carotenoid spectrophotometric methods, respectively. In order to determine the total protein of the novel sauce, as described by Pedrol et al. [14], 10 μL of the sample and 180 μL of Bradford working solution was placed in 96-well plate wells, followed by absorbance measurement at 595 nm after 5 min. The total phenolic content of the functional sauce was determined by the Folin–Ciocalteu method, as described by Ainsworth et al. [15]. A total 50 μL of the sample, 20 μL of 7.5% Na_2_CO_3_ solution, and 20 μL of Folin–Ciocalteu reagent were added in the wells of a 96-well plate, and the absorbance was then measured at 765 nm after 30 min in a dark environment. Total functional sauce carotenoids were measured by simple spectrophotometric methods at 450 nm after 30 min in the dark, while total antioxidant capacity was determined by the ferric reducing antioxidant power (FRAP) method, as described by Argyri et al. [16], adding 20 μL of novel sauce and 80 μL of FRAP reagent in 96-well plate wells, and then measuring its absorbance at 595 nm after 30 min in the dark. Τhe results were quantified using standard curves.

### 2.5. Study Design

The experimental design included a randomized, single-blinded, interventional study with a crossover design involving two trial periods, separated by a 1-week washout period. The study was conducted at the Human Nutrition Unit in the Laboratory of Nutrition and Public Health, Limnos, Greece.

**Figure 2:**
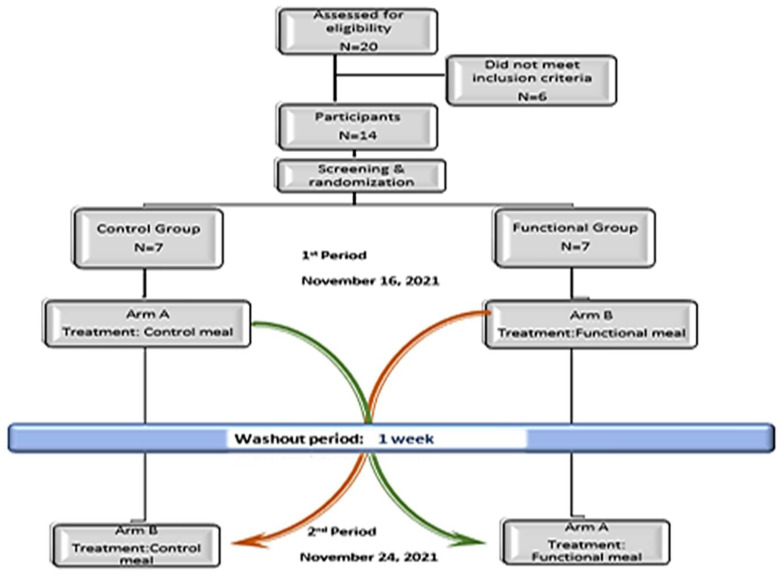
Study design illustration.

The participants were randomly assigned to the control or functional group, and they crossed over from one arm of the study to the other. Individuals who joined the control group, during each test period, received the control meal, and those who joined the functional group received the functional meal. Figure 2 shortly describes the study design with an illustration.

All subjects arrived at 9 a.m. at the Nutrition Unit. Before each experimental period, participants followed a 12 h fast and abstinence from dietary supplements and any medication. Abstinence from foods high in antioxidants and alcohol consumption for 24 h before the start of the trial periods was also requested. After confirmation of the implementation of the above instructions, the volunteers were asked to complete a short, self-administrated 24 h recall questionnaire, which recorded all meals consumed in the last 24 h.

They were then offered a meal containing white rice, butter, cheese, and sauce (258 g), while a glass of water (250 mL) was available to each participant. In the morning before each visit, the researchers prepared 7 control and 7 functional meals, dependent on the randomization, in white dishes and labelled them with the participant’s ID. This procedure was conducted to avoid any kind of bias from the participants by knowing the test meal before consumption.

### 2.6. Blood Sampling and Analyses

After an overnight fast, ten mL of blood sample (baseline) was withdrawn from all volunteers. The subjects ingested the test meal within 15 min, and postprandial blood samples were taken at 30 min, 1.5 h, and 3 h after completion of the test meal. Blood samples were collected in EDTA and citric acid vacutainers for plasma separation or in heparin vacutainers for serum separation. Each volunteers’ plasma and serum for each time of sampling were immediately separated by centrifugation at 20,000× *g* for 10 min at 4 °C in a tabletop high-speed refrigerated centrifuge (Thermo Scientific ST16R, Thermo Fisher Scientific, Waltham, MA, USA). Plasma and serum were then aliquoted and stored at −40 °C at the research facility (Laboratory Freezer, MRC Laboratory Instruments, Holon, Israel).

Once the study was completed, serum samples were analyzed with an automated biochemical analyzer (COBAS c111, Roche, Basel, Switzerland) for total HDL- and LDL-cholesterol, glucose, triglycerides, and uric acid determination.

Total plasma antioxidant capacity (TAC) was evaluated by FRAP assay, as described by Argyri et al. [16,17]. Inhibition of platelet-activating factor (PAF)-induced thrombosis in platelet-rich plasma was determined according to the method of Antonopoulou et al. [18].

### 2.7. Data Analysis

Sample size calculation was performed using G*Power software version 3.1.9.2. Considering a probability of 95% that the study will detect a treatment difference at a two-sided 0.01 significance level, the sample of 10 individuals allows the detection of a difference of 0.28 mmol TAC/L between the control group and the intervention group, calculated from the expected SD = 0.2 between the differences of the meal groups. All statistical analyses were performed using SPSS 21.0 for Windows (IBM Corporation, New York, NY, USA). Data are reported as mean ± standard deviation (SD). Statistical significance was accepted at *p* ≤ 0.05. All data were taken into account and assessed for normal distribution employing the Kolmogorov–Smirnov normality test. Serum total, HDL- and LDL-cholesterol, glucose, triglycerides, uric acid, plasma antioxidant activity (TAC), and aggregation variables were analyzed via repeated ANOVA measures, with Geisser–Greenhouse correction. Post-hoc tests were performed via the Bonferroni test. Changes in the clinical characteristics from baseline to follow-up (within-group variation) were calculated using paired samples *t*-test (two tailed). The incremental area under the curve (iAUC) for significant postprandial treatment × time interactions was calculated by using the trapezoidal rule integrated count areas above and below the fasting baseline concentration. Paired samples t-tests confirmed significant differences in the results from iAUC (*p* ≤ 0.05).

## 3. Results

### 3.1. Functional Sauce In Vitro Measurements

The novel miso-type sauce provided 0.16 mg of total proteins per gram, 57.6 mg total phenolics per gram, expressed as gallic acid equivalents, 48.16 mg of total carotenoids per gram, expressed as β-carotene equivalents, and 165.18 mmol FeSO_4_/g of total antioxidant activity.

### 3.2. Baseline Characteristics

Twenty participants were assessed for eligibility; however, only fourteen were randomized into the treatments, and six subjects were excluded because they did not met the inclusion criteria. The mean age and BMI were 23.5 ± 2.7 of age and 24.5 ± 3.5 kg/m^2^, respectively. The baseline characteristics of the participants are described in Table 2.

### 3.3. Postprandial Plasma Total Antioxidant Capacity (TAC)

**Figure 3:**
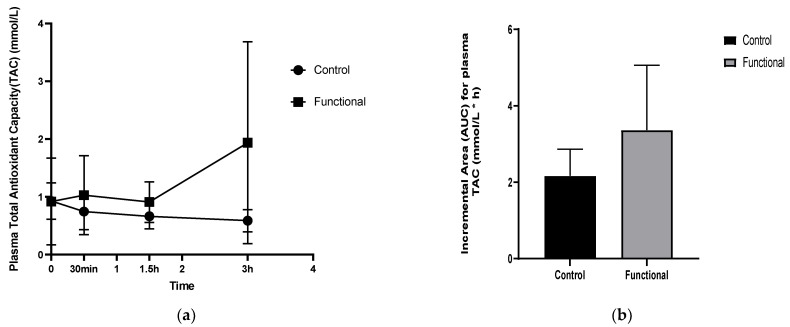
(**a**) Incremental changes in postprandial plasma total antioxidant capacity (TAC). (**b**) The incremental area under the curve (iAUC) for postprandial plasma TAC.

The incremental changes in postprandial plasma TAC after consumption of the experimental meals are demonstrated in Figure 3. A significant treatment × time interaction (*p* = 0.0009) and a group effect (*p* = 0.03) were found for plasma TAC. Postprandial total antioxidant capacity following the functional meal, containing the novel miso-type sauce, was significantly higher than following the control meal at 3 h (*p* = 0.015, MD = 0.83), as shown in Figure 3a. A significant increase in plasma TAC (*p* = 0.05) 3 h after the functional meal intake was observed, while a significant decrease was found 3 h after control consumption (*p* = 0.0008). The iAUC of plasma TAC for the functional group presented as significantly increased (*p* = 0.035) relative to the control meal (Figure 3b).

### 3.4. Postprandial Serum Lipids, Glucose and Uric Acid

**Figure 4:**
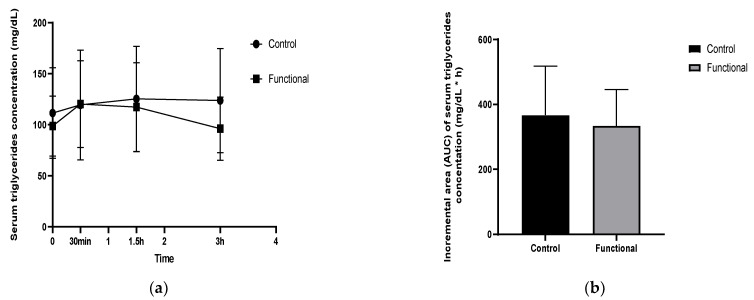
(**a**) Incremental changes in postprandial serum triglycerides (TG) concentration. (**b**) The incremental area under the curve (iAUC) for postprandial serum TG.

Concerning serum lipids, a significant treatment × time interaction (*p* = 0.0009) and a time effect (0.0015) were observed for serum triglycerides concentrations. The functional meal attenuated significantly (*p* = 0.003, MD= −20.3) the increase in TG compared to the control meal, in the last 1.5 h (1.5–3 h), as shown in Figure 4a. The results show that consumption of the innovative miso-type sauce resulted in a 0.9-fold statistically significant lower iAUC value for serum triglycerides level (*p* = 0.03) when compared with the control group (Figure 4b).

**Figure 5:**
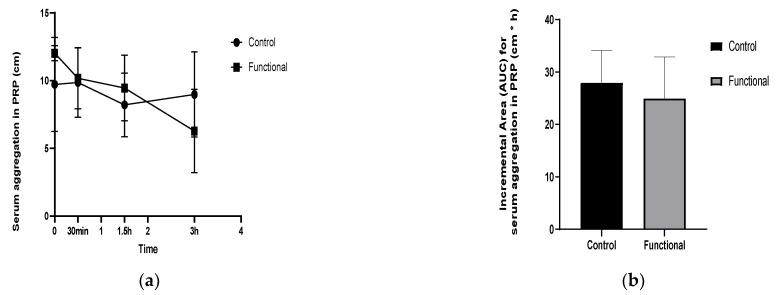
(**a**) Incremental changes in aggregation in PRP (cm). (**b**) The incremental area under the curve (iAUC) for aggregation in PRP (cm × h).

A significant treatment × time interaction was found for platelet aggregation in platelet-rich plasma (PRP) (*p* = 0.037). The functional meal significantly reduced platelet aggregation 30 min after consumption (*p* = 0.023) compared to the control meal and a significant change from the baseline was observed at 3 h after the novel miso-type sauce intake (*p* = 0.018). Non-significant responses were observed for the control meal (Figure 5a). A significant (*p* = 0.04) reduction in the iAUC of postprandial PRP aggregation (2.57) was perceived following consumption of the functional miso-type sauce compared with the control sauce, as shown in Figure 5b.

**Figure 6:**
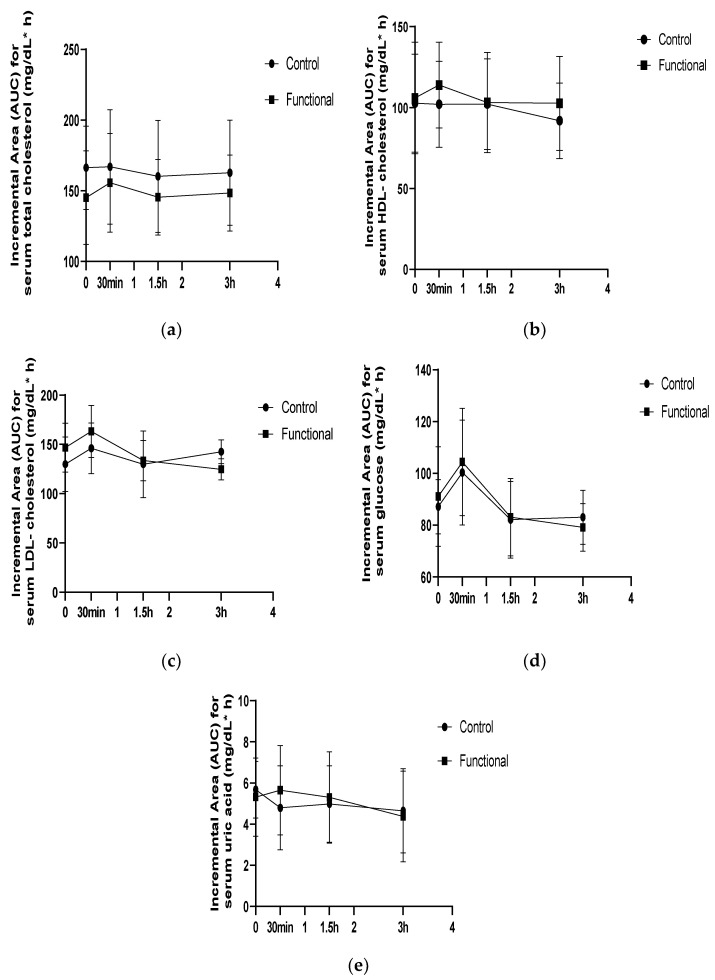
(**a**) Incremental changes in postprandial total cholesterol concentration. (**b**) Incremental changes in postprandial HDL-cholesterol concentration. (**c**) Incremental changes in postprandial LDL-cholesterol concentration. (**d**) Incremental changes in postprandial serum glucose concentration. (**e**) Incremental changes in postprandial uric acid concentration.

A time effect was detected for postprandial LDL-cholesterol levels (*p* = 0.02), but no one treatment × time interaction was found. The LDL-cholesterol levels were significantly reduced 3h after the functional meal consumption (*p* = 0.043) when compared to the baseline, while a non-significant raise was found 3 h after the control meal compared to the baseline concentration. No statistically significant interactions or differences were observed regarding the remaining biomarkers tested (total cholesterol, HDL-cholesterol, and uric acid), as there was a similar response to the levels of these biomarkers after consuming both meals. The postprandial changes in serum total, HDL- and LDL- cholesterol, glucose, and uric acid are presented in Figure 6.

Table 3 summarizes postprandial responses of serum lipids, glucose, aggregation, and plasma TAC, following a high-carbohydrate and fat meal, containing a novel miso-type or a control sauce.

## 4. Discussion

Miso has received considerable attention as a potential approach to reduce the risk of chronic illness, such as cardiovascular disease, and its long-term consumption has also been associated with a possible lower risk of mortality [19]. Recent studies have reported the possible utilization of food by-products, such as grain brans, and their solid-state fermentation by fungus, in order to develop functional foods, enhanced in bioactive compounds [20]. The fungal fermentation of legume-based foods in cereal bran substrates has been suggested as it may enhance the phenolic and carotenoids bioavailability, leading to increased antioxidant activity and exhibiting protective effects against cardiovascular disease [21]. Moreover, it has been reported that the utilization of carotenoids recovered from fruit by-products (e.g., peels, pomaces) may be used for novel, functional food production [22]. Carotenoid-rich fruit peels, such as orange and carrot peels, possess prominent functional and antioxidant properties which are potentially effective for human digestion and metabolism, cholesterol and triglyceride homeostasis, and diabetes management [23]. In a previous study, the consumption of a spread cheese, enhanced with mountain tea and orange peel extract, led to increased postprandial total antioxidant activity in the plasma of healthy volunteers [24]. This was the first clinical study–nutritional intervention to investigate the acute effect of a novel, fermented miso-type sauce, based on legumes and enhanced with carotenoids from fruit peels extracts, on postprandial serum lipids, glucose, aggregation, and plasma total antioxidant capacity (TAC) levels.

Post-meal glycemic metabolism imbalance and hyperlipidemia occurs after a meal rich in fats and carbohydrates, leading to the overproduction of reactive oxygen species (ROS). Excessive ROS formation appears to be an important factor in the development of cardiovascular disease [25]. It has been reported that dietary antioxidants intake may reduce oxidative stress, helping normal physiological function maintenance [5]. Certain phytonutrients, such as polyphenols and carotenoids, may suppress postprandial oxidative stress markers in the blood, which are generated transiently from various tissues and cells after the consumption of a meal high in fat and carbohydrates [26].

In the present study, acute consumption of the functional miso-type sauce, enhanced with carotenoids from fruit peel extracts, led to increased plasma TAC 3 h after functional meal intake, when plasma TAC 3 h after the control sauce intake was significantly decreased. These findings are in accordance with our previous study, where we found an increase in plasma antioxidant capacity 3 h after the intake of a meal high in fat and carbohydrates, containing a novel spread cheese, enhanced with a mountain tea and orange peel extract [24]. Similar results were reported by Urquiaga et al. [27], who, in a randomized nutritional intervention, studied the postprandial effect of a high-fat meal, consumed in combination with an antioxidant-rich berry concentrate, and observed an increase in malondialdehyde (MDA), increase in plasma antioxidant activity, and decrease in protein carbonyls. Moreover, as mentioned by Laus et al. [28], the acute consumption of functional bran pasta enriched with lipophilic antioxidants (e.g., carotenoids), improved postprandial serum antioxidant status, and also the intake of functional bran pasta enriched with phenolics beneficially effected postprandial serum antioxidant status.

The increased postprandial plasma antioxidant capacity may be a synergistic result of the natural antioxidants that the novel sauce contained. The fruit peels’ (carrot, orange, banana, kiwi, apple, watermelon) extracts, which was used for the enhancement of the functional miso-type sauce, provide bioactive phenolic compounds, carotenoids, flavonoids, phytosterols, etc., contributing to the overall antioxidant activity. Postprandially, these bioactive compounds are bioavailable and are able to acutely increase plasma antioxidant capacity, reducing oxidative stress [29]. In their review, Mirmiran et al. [30] concluded that several bioactive compounds, derived from fruit and vegetable by-products (carrot, apple, orange peel, etc.), may contribute to oxidative stress attenuation, increasing total plasma antioxidant capacity [8,30]. A suggested mechanism by which carotenoids may regulate oxidative stress is their possible transport facilitation to the nucleus via the Nrf-2 pathway by phase II enzymes and glutathione-S-transferases activation [31]. Moreover, β-carotene, which is the main carotenoid found on the fruit peel extract, used for the miso-type sauce enhancement, has been reported for its beneficial role in regulating the eNOS expression and molecules adhesion via the activation of Ca^2+^/calmodulin-dependent protein kinase II (CaMKII) pathway in an in vitro model of endothelial dysfunction induced by IL-1β [32]. Furthermore, the antioxidant properties of fermented chickpeas in the functional miso-type sauce, may be associated with the presence of isoflavones, which have been reported as antioxidant enzyme modulators [33]. It is notable that the fermentation by *Aspergillus oryzae* may lead to the enhanced production of antioxidant compounds, such as carotenoids, contributing to the overall plasma antioxidant activity [15,18].

Another key finding of this study is that antioxidant-rich miso-type sauce, taken as a part of a rich in fat and carbohydrates meal, mitigated postprandial triglycerides increase 3 h after meal consumption. It is notable that a decrease of LDL-cholesterol levels at 3 h, when compared with baseline concentrations, was found after the functional miso-type sauce. This possible beneficial effect of the novel sauce on postprandial triglyceride and LDL-cholesterol levels may reflect the lipid-lowering properties of phytonutrients, dietary fiber, and certain peptides. Dietary polyphenols have been suggested as pancreatic lipase inhibitors and they may reduce the digestion and absorption of triglycerides [34]. The mechanism underlying the ability of polyphenols to reduce blood lipid levels involves the improvement of insulin resistance, protection against oxidation, and free radical scavenging [35]. Heterogeneity has been noticed about the acute effect of the phenolic supplementation of high-fat meals, as the results may be affected by more factors, such as fat meal composition, its energy density, and the intervention length. However, in several clinical trials, polyphenol metabolites such as quercetin dehydrate and resveratrol seem to significantly attenuate the rise of postprandial TG or Apo B-48/100 production rates [36]. A study examined the acute effect of polyphenol-rich cocoa in the framework of a high-fat meal, in overweight and normal-weight adults, and found that the phenolic supplementation of the meal with epicatechin attenuated postprandial triglycerides and lipid oxidation 4 h after the meal [37].

Dietary fiber provided by the functional miso-sauce from cereals and legumes may exert a beneficial role on the triglyceride-lowering effect, which could be attributed to micellization process disruption at the intestinal level. In this way, dietary fibers contribute to gut motility, preventing fat absorption through the formation of a water barrier between nutrients and the intestinal mucosa, and regulating the intestinal microflora. An alternative way of dietary fibers’ beneficial action is related to the chylomicron secretion alteration [38]. Additionally, it has been mentioned by Shi et al. that chickpeas’ peptides act as hypolipidemic agents, lowering serum triglycerides and total and LDL-cholesterol. Chickpeas’ peptides may inhibit the fatty acid synthetase and 3-hydroxy-3-methyl-glutaryl-CoA reductase action, altering the peroxisome activated by the multiplier receptors and LDL-receptor expression [39]. Concerning the LDL-cholesterol decrease observed, similar results have been revealed by Lim et al. [40], who found that a traditional red pepper paste (Kochujang), fermented by *Aspergillus oryzae*, reduced total cholesterol levels and low-density lipoprotein-C (LDL-C) in hypercholesterolemic subjects. It has been noticed that fermented foods may exert hypolipidemic effects as good sources of bioactive nutrients such as dietary fiber, natural antioxidants, and phytochemicals. It is noteworthy that several studies suggest wheat bran, which is used as a fermentation substrate during the functional miso-type sauce production, as a potential functional component for cardiovascular protection, as it may inhibit platelet activity and aggregation, and also is able to decrease triglyceride synthesis, due to its alkylresorcinol content [34].

The enhancement of foods with bioactive components, derived from food by-products, in order to produce innovative, functional foods remains a viable approach to treating cardiovascular and metabolic risk factors, such as postprandial hyperlipidemia, hyperglycemia, and oxidative stress. However, combinations of bioactive compounds from plant-based functional foods and food by-products may exhibit synergistic hypolipidemic and antioxidant effects, thereby yielding more potent protective activity [37]. Due to the phenomenon of “synergy” being a multifactorial process, involving many mechanisms in order to understand the exact postprandial complex effect of each bioactive ingredient on the plasma antioxidant status, and also to lipid and glycemic profiles, the evaluation of each bioactive should be examined extensively. Furthermore, the blood concentration of combined metabolites of dietary phytochemicals, such as polyphenols and carotenoids, found in the innovative, functional sauce, may have a minimal impact on the plasma total antioxidant capacity. Thus, the measurement of each one of these bioactive components individually is recommended in order to achieve a quantitative estimation of postprandial changes and this is a possible limitation of the present study. Alternatively, it may be appropriate to measure specific biomarkers of oxidative stress [36,41]. Although the study detected statistical significance differences in several biomarkers, the inter-person physiological variations due to the relatively small number of participants, as an extra limitation of the study, lead to the conclusion that more clinical and epidemiological prospective studies are needed to exact safer results.

Finally, there is a need for the establishment of new approaches, in order to provide better scientific evidence about the acute effect of novel functional foods on postprandial biomarkers of inflammation and oxidative stress. Of interest is nutrigenomics, including proteomics and metabolomics, providing more details about the physiological state from phenotype examination. In particular, metabolomics may provide information about the metabolic pathways involved in response to functional meals intake [42].

## 5. Conclusions

The present study demonstrates that the consumption of a novel, fermented, functional miso-type sauce, based on legumes and enhanced with bio-carotenoids from fruit peels’ extract, acutely increased the total antioxidant capacity of plasma, with a parallel reduction of serum triglycerides, LDL-cholesterol, and aggregation 3 h after intake. This study highlighted the possible beneficial acute effect of a functional fermented sauce, enhanced with bioactive compounds from food by-products, on postprandial biomarkers and biomolecules of oxidative stress and lipemia; thus, the inclusion of such a novel, functional sauce in the context of a balanced diet may contribute to cardiovascular disease prevention, as a promising aspect. Further investigation about the long-term effect of the novel miso-type sauce is needed, in both healthy volunteers and subjects with high cardiovascular risk, in order to achieve safer conclusions.

## Figures and Tables

**Table 1:** Nutritional value of the experimental meals (each meal).

Meals’ Nutritional Value		
	Control	Functional
Energy (kcal)	932.155	932.155
Carbohydrates (g)	140.945	140.945
Fat, total (g)	29.972	29.972
Protein (g)	29.295	29.298
Saturated fat (g)	17.6935	17.6935
Unsaturated fat (g)	8.6685	8.6685
Cholesterol (mg)	73.6	73.6
Dietary fiber, total (g)	4.6	4.6
Sugar, total (g)	0.845	0.845
Total phenolics (mg/g)	0	57.6
Total carotenoids (mg/g)	0	48.16
Total antioxidants (mmol/g)	0	165.18

**Table 2:** The baseline characteristics of participants.

Variable	
General Characteristics	N
Volunteers	14
Men	6
Women	8
Smoking	4
Physical Activity	
Low	2
Medium	6
High	6
	Mean ± SD
Age (years)	23.5 ± 2.7 ^a^
Anthropometry and Body Composition	
Weight (kg)	71.5 ± 15.3 ^b^
Height (cm)	170.1 ± 8.1 ^c^
BMI	24.5 ± 3.5 ^d^
Fat mass (kg)	20.9 ± 7
Muscle mass (kg)	53.4 ± 11.1
Body water (kg)	55 ± 6.8
Bone mass (kg)	2.8 ± 0.3
Waist/hip circumference ratio	0.7 ± 0.17

Different letters indicate statistical signifficant differences.

**Table 3:** Postprandial responses of serum lipids, glucose, aggregation, and plasma TAC, following a high-carbohydrate and fat meal, containing a novel miso-type or a control sauce.

	Control	Functional
Total cholesterol at baseline (mg/dL)	170.53 ± 34.5	145.15 ± 33.2
Δ 30 min	−0.61 ± 0.43	10.53 ± 7.45
Δ 1.5 h	−10.28 ± 7.27	−10.3 ± 7.28
Δ 3 h	3.16 ± 2.23	2.07 ± 2.17
iAUC	408.9 ± 180.78	446.16 ± 81.15
Glucose at baseline (mg/dL)	87.9 ± 10.77	91.07 ± 19.2
Δ 30 min	13.18 ± 9.32	13.38 ± 9.46
Δ 1.5 h	−18.18 ± 12.85	−21.37 ± 15.11
Δ 3 h	0.9 ± 0.64	−3.91 ± 2.76
iAUC	262.13 ± 37.7	246.7 ± 66.59
Triglycerides at baseline (mg/dL)	111.61 ± 44.48	98.76 ± 29.4
Δ 30 min	7.84 ± 5.54	21.61 ± 15.28
Δ 1.5 h	5.92 ± 4.18	−3.07 ± 2.17
Δ 3 h	−12.5 ± 8.86	−20.9 ± 10.55
iAUC	412.61 ± 152.08	298.27 ± 112.07
HDL-cholesterol at baseline (mg/dL)	98.12 ± 28.78	98.57 ± 31.36
Δ 30 min	−0.76 ± 0.47	21.61 ± 15.28
Δ 1.5 h	0.13 ± 0.09	−3.07 ± 2.17
Δ 3 h	−10.37 ± 7.33	−17.9 ± 10.55
iAUC	270.61 ± 100,47	276.68 ± 97.71
LDL-cholesterol at baseline (mg/dL)	129.16 ± 24.11	145.36 ± 25.91
Δ 30 min	18.64 ± 13.18	−12.72 ± 8.99
Δ 1.5 h	−18.68 ± 13.21	−0.41 ± 0.29
Δ 3 h	12.73 ± 9	−9.89 ± 6.22
iAUC	422.18 ± 39.36	432.65 ± 57.76
Uric acid at baseline (mg/dL)	5.68 ± 1.37	5.44 ± 1.84
Δ 30 min	−0.88 ± 0.62	0.33 ± 0.23
Δ 1.5 h	0.19 ± 0.13	−0.35 ± 0.24
Δ 3 h	−0.33 ± 0.23	−0.9 ± 0.65
iAUC	14.74 ± 5.48	15.47 ± 4.54
Total plasma antioxidant capacity (TAC) (mmol/L)	0.93 ± 0.31	0.91 ± 0.74
Δ 30 min	0.23 ± 0.16	0.18 ± 0.12
Δ 1.5 h	−0.55 ± 0.32	−0.27 ± 0.19
Δ 3 h	−0.09 ± 0	0.7 ± 0.42
iAUC	2.06 ± 0.69	3.46 ± 1.69
Aggregation in PRP (cm)	10.01 ± 3.3	0.82 ± 4.18
Δ 30 min	0.05 ± 0.03	−2.55 ± 1.8
Δ 1.5 h	−1.37 ± 0.97	1.82 ± 1.29
Δ 3 h	−0.02 ± 0.06	−2.7 ± 1.2
iAUC	29 ± 6.08	23.98 ± 7.87

## Data Availability

The data presented in this study are available within this article.
